# Traditional Chinese medicine’s holistic approach: regulating microglia-driven neuroinflammation for the resolution of Alzheimer’s disease

**DOI:** 10.3389/fncel.2025.1691253

**Published:** 2025-11-03

**Authors:** Jialing Yu, Xinya Bao, Chuchu Shan, Zebin Yu, Yajie Yu, Hongying Wang, Yuyan Zhang

**Affiliations:** 1 School of Life Sciences, Zhejiang Chinese Medical University, Hangzhou, Zhejiang, China; 2 Jiaxing Hospital of Traditional Chinese Medicine, Zhejiang Chinese Medical University, Jiaxing, Zhejiang, China; 3 Lanxi People’s Hospital, Jinhua, Zhejiang, China

**Keywords:** Alzheimer’s disease, microglia, neuroinflammation, traditional Chinese medicine, polarization

## Abstract

**Background:**

Alzheimer’s disease (AD) is a progressive neurodegenerative disease characterized by cognitive dysfunction, motor abnormalities, and memory disorders, with a persistently high and rising incidence. The pathological features of AD include the extracellular deposition of the amyloid beta peptide (Aβ), the accumulation of neurofibrillary tangles (NFTs), and neuroinflammation. Microglia (MG), the main immune cells in the central nervous system (CNS), can transform into different phenotypes. An imbalance in their phenotypic transformation may induce neuroinflammation and lead to neurological diseases, playing a central role in the onset and progression of AD.

**Purpose:**

This article aims to briefly review the key role of microglia-mediated neuroinflammation in the pathogenesis of AD and to summarize and analyze the strategies of traditional Chinese medicine (TCM) for targeting microglia in AD treatment.

**Methods:**

Literature review and analysis were conducted to summarize the role of microglia-mediated neuroinflammation in AD pathogenesis and to collate TCM therapeutic strategies aimed at modulating microglia.

**Results and conclusion:**

Microglia-mediated neuroinflammation plays a central role in the pathological progression of AD. TCM demonstrates potential in intervening in AD neuroinflammation by regulating the microglial phenotype and function. These related therapeutic strategies warrant further summary and analysis.

## 1 Introduction

Alzheimer’s disease (AD), a progressive neurodegenerative disease characterized by cognitive dysfunction, motor abnormalities, and memory disorders, has a high and persistently increasing incidence, accounting for 60 to 70% of global dementia cases (Wang et al., 2020). It is now recognized that Alzheimer’s disease has three key pathological features: the extracellular deposition of amyloid beta peptide (Aβ), the accumulation of neurofibrillary tangles (NFTs), and neuroinflammation. Research into the changes in the levels of inflammatory markers in AD patients, along with the identification of AD risk genes associated with innate immune function, has revealed that the amyloid deposition hypothesis is insufficient to explain many aspects of the pathogenesis of AD and that neuroinflammation plays an important role in the pathogenesis of AD (Leng and Edison, 2021). Persistent neuroinflammation mediated by glial cells is an important cause of neurodegenerative pathology and cognitive decline in AD (Dhapola et al., 2021).

Neuroinflammation refers to the inflammatory response caused by the accumulation of neuroglial cells in the central nervous system (CNS) as a reaction to injury, with microglia (MG) being considered the key factor in mediating the inflammatory response. Phenotypic changes in MG from a normal physiological state (M0) to a stimulated state (M1/M2) affect inflammatory mediator release. An overactivated M1 phenotype exacerbates the inflammatory response and may induce neurological disorders, whereas the M2 phenotype exerts neuroprotective effects through phagocytosis, regulation of anti-inflammatory factor expression, and promotion of damaged neuron repair.

With the in-depth study of the mechanism of action of traditional Chinese medicine (TCM), the mechanism of action of the active ingredients of TCM has gradually become clearer, and research on TCM in the field of AD has gradually gained emphasis, offering new therapeutic hope for patients. In the theoretical system of TCM, AD is categorized under “dementia” and “forgetfulness” and is considered to be related to the brain and closely associated with the kidneys, heart, liver, spleen, and other organs. The fundamental pathogenesis is characterized by a deficiency in the root and an excess in the branches. In clinical practice, TCM often utilizes herbs that are categorized as tonifying, blood-activating, and phlegm-removing to improve cerebral blood circulation and reduce neuroinflammatory responses, thereby achieving the goal of treating and preventing AD. Medicinal herbs, such as *Sophora flavescens* (Li et al., 2020), *Epimedium* (Wang et al., 2019), and Rehmannia(Choi, 2019), as well as compound prescriptions such as Hei Xiao Yao San (Lou et al., 2019) and Nao Ling Tang (Ge, 2013), have demonstrated significant effects in targeting MG to improve clinical symptoms of AD. Experimental studies have shown that TCM can effectively alleviate Alzheimer’s disease caused by MG-mediated neuroinflammation by inhibiting M1-type activation, promoting M2-type polarization, and regulating the expression of MG receptor proteins through its holistic and dynamic regulatory features, as well as multi-component, multi-pathway, and multi-target mechanisms. We review the phenotype and transformation of MG, the role of MG-mediated neuroinflammation in promoting the occurrence and development of Alzheimer’s disease, and the mechanisms through which TCM regulates MG-mediated neuroinflammation to prevent and treat AD. It explores the therapeutic effects of TCM in the prevention and treatment of AD, providing a reference for the development of new Chinese medicinal drugs with proven efficacy and improving clinical outcomes for the disease.

## 2 Methods

A systematic literature search was conducted in the following electronic databases from January 2000 to June 2025: PubMed, Web of Science Core Collection, and the China National Knowledge Infrastructure (CNKI). The search strategy utilized a combination of Medical Subject Headings (MeSH) terms and free-text keywords related to three core concepts: (1) Alzheimer’s disease, (2) microglia and neuroinflammation, and (3) TCM. The specific search string for PubMed was as follows: (“Alzheimer Disease” OR “Alzheimer’s Disease”) AND (“Microglia” OR “Neuroinflammation” OR “Microglial Polarization”) AND (“Medicine, Chinese Traditional” OR “Traditional Chinese Medicine” OR “Herbal Medicine” OR “Chinese Herbs” OR specific herb names such as “Ginseng” OR “*Ginkgo biloba*”).

Similar strategies were adapted for the syntax requirements of other databases (Web of Science and CNKI). Additionally, the reference lists of the retrieved relevant review articles and primary studies were manually screened to identify any potentially eligible publications that might have been missed in the database search.

## 3 The central role of microglial polarization regulation in Alzheimer’s disease

AD, characterized by cognitive dysfunction, motor abnormalities, and memory disorders, is a progressive neurodegenerative disease. Its development is divided into three stages: preclinical, mild cognitive impairment (MCI), and dementia. Alzheimer’s disease has three key pathological features: the extracellular deposition of Aβ, the accumulation of NFTs throughout the brain, and neuroinflammation. The accuracy of Aβ as a biomarker for AD in the amyloid hypothesis proposed two decades ago has been seriously challenged. Studies have found Aβ in approximately 30% of cognitively normal older individuals, and the strategy of clearing Aβ to treat AD has also been disappointing, indicating that the “amyloid cascade hypothesis” cannot fully explain the molecular mechanisms of AD pathogenesis. In addition to amyloid hypothesis, oxidative stress hypothesis, cholinergic hypothesis, and neuroinflammation hypothesis provide multiple angles for studying the pathogenesis of AD. Among these hypotheses, neuroinflammation has been proven to participate in the entire process of AD. An increasing number of studies have shown that sustained neuroglia-mediated neuroinflammation is a significant contributor to the neurodegenerative process and cognitive deficits in AD. Studies have also found that neuroinflammation interacts with AD, and the role of MG-mediated neuroinflammation in the occurrence of AD should be further explored (Wang et al., 2023).

MG, myeloid innate immune cells in the central nervous system (CNS), can transform into different phenotypes through different states of the organism and play important roles in immune surveillance, inflammatory response, neuroprotection, neurotransmitter regulation, and neuroendocrinology. However, over-activated M1-type MG may induce neuroinflammation, leading to the onset and progression of neurological diseases such as AD.

### 3.1 Phenotypes

MG, as multipotent cells, are divided into “resting MG” (M0) and “activated MG,” the latter of which is divided into M1 pro-inflammatory and M2 anti-inflammatory phenotypes. The M2 type of MG can be further divided into M2a, M2b, and M2c—three subtypes with different activation states. M2a-type MG are selectively activated and promote immune elimination and tissue regeneration; M2b-type MG are activated by immune complexes mediated through Fcγ receptors and secrete IL-10; M2c-type MG arise from the deactivation of M1-type MG under the influence of glucocorticoids or IL-10, secreting TGF-*β* and sphingomyelinase (Wang et al., 2018; Hu and Hua, 2018).

### 3.2 Transformation

The phenotype transformation of MG is regulated by related signal transduction and growth factors. Under resting conditions, MG are of the M0 phenotype. MG maintain cellular environmental homeostasis by activating key cytokine signaling axes of neurons and astrocytes (AST), playing roles in immune surveillance, defense, and tissue repair (Ransohoff and Perry, 2009).

When brain tissue is damaged, MG are activated into M1 or M2 types according to different related signal transduction and growth factors, protecting the CNS from neural damage or pathogenic invasion and subsequent neuroinflammatory responses (Freilich et al., 2013), thereby jointly maintaining the balance of the CNS microenvironment (Tang and Le, 2016; Chio et al., 2015).

#### 3.2.1 M1 polarization drives neuroinflammation

M1 is a pro-inflammatory phenotype: lipopolysaccharide (LPS) or the water-soluble dimeric cytokine interferon-*γ* (IFN-γ) stimulates the formation of M1-type MG, a process also known as “classical activation.” Studies have shown that M1-type MG can promote the release of various inflammatory factors, such as tumor necrosis factor (TNF-*α*), interleukin-1β (IL-1β), interleukin-6 (IL-6), and nitric oxide (NO), inducing neuronal damage and oxidative stress, leading to synaptic loss, and accelerating disease progression. Its biomarkers are mainly TNF-α, IL-1β, IL-6, IL-12, and cluster of differentiation 16/32 (CD16/32) (Chen et al., 2024; Li et al., 2019).

#### 3.2.2 M2 polarization repairs the function

M2 is an anti-inflammatory phenotype: IL-4 promotes the formation of the M2 phenotype, also known as “alternative activation,” which has various immune protective functions. It can produce anti-inflammatory factors and neuroprotective factors, such as interleukin-10 (IL-10), interleukin-4 (IL-4), brain-derived neurotrophic factor (BDNF), and insulin-like growth factor 1 (IGF-1), to enhance the phagocytic action of MG on Aβ, playing an immune elimination role in the brain to suppress inflammation, and to promote the repair and regeneration of CNS tissue, improving disease progression. Its biomarkers are essentially IL-10, Arg-10, and CD206 (Li et al., 2019; Liang et al., 2017; Holden et al., 2014). Studies have found that M2 microglia are further divided into three subtypes: M2a, mainly involved in repair and regeneration, with Arg-1 as the primary biofactor marker; M2b, related to immune regulation, with suppressor of cytokine signaling 3 (SOCS3) as the primary biofactor marker; and M2c, involved in neuroprotection and the release of some anti-inflammatory cytokines, with Cluster of Differentiation 163 (CD163) as the primary biofactor marker (Walker and Lue, 2015).

**Figure 1:**
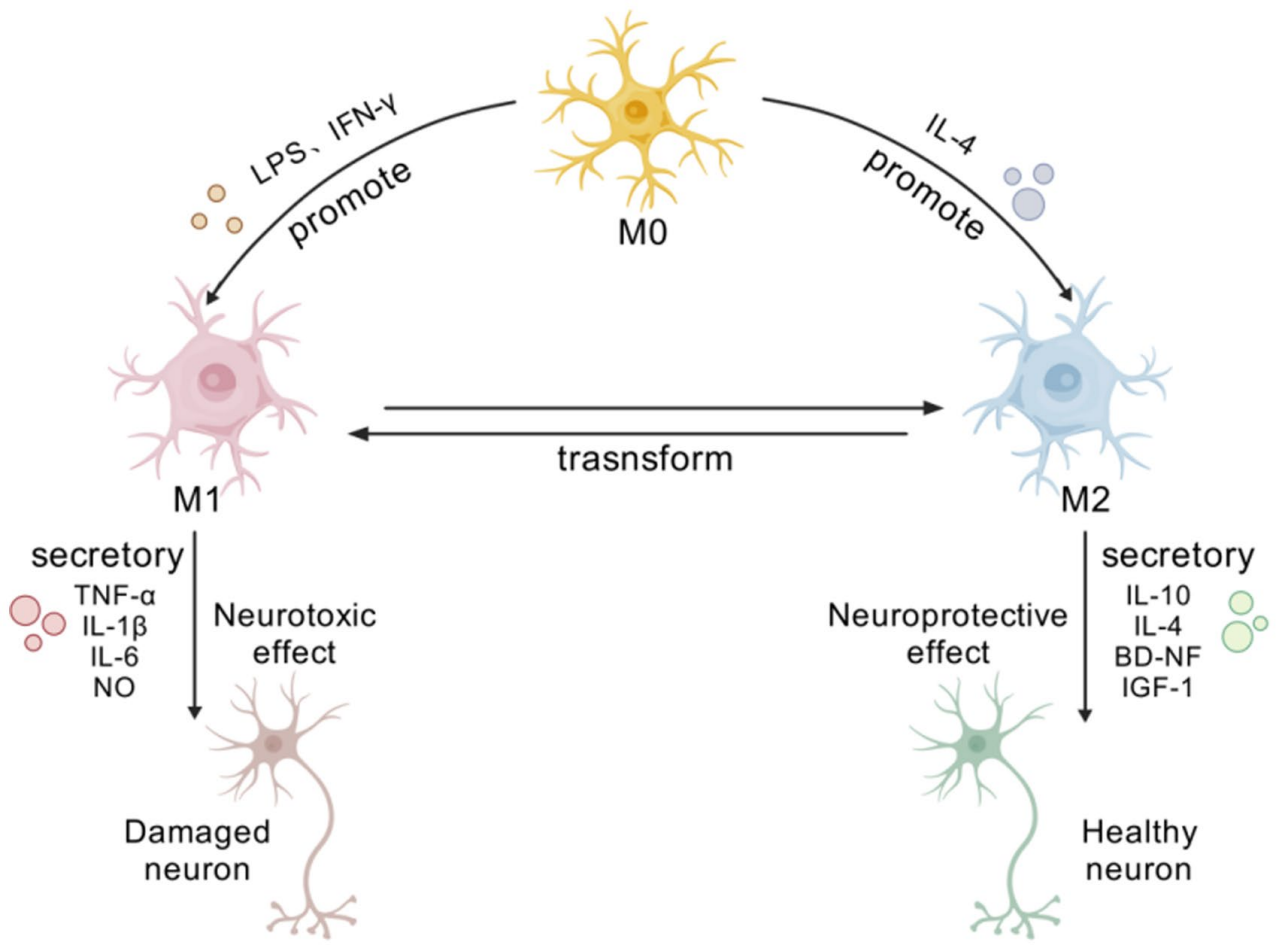
Phenotypic transformation and functional diagram of MG. [Created with BioGDP.com (Jiang et al., 2025)] At rest, microglia exhibit an M0 phenotype. Upon brain injury, they become activated and polarize into M1 or M2 phenotypes due to diverse signaling pathways and growth factors. M1 microglia adopt a pro-inflammatory phenotype, releasing various pro-inflammatory mediators, such as TNF-*α*, IL-1β, IL-6, and NO. These factors induce neuronal damage and oxidative stress, accelerating disease progression. M2-type cells secrete various anti-inflammatory and neuroprotective factors, including IL-10, IL-4, BDNF, and IGF-1, and possess diverse immunoprotective functions.

Furthermore, studies have found through *in vitro* cell culture that the characteristic markers of the M1 and M2 phenotypes of MG increase over time and the proportion of different phenotypes also undergoes dynamic changes. In the process of maintaining neurostasis, the initial M2 type is stronger than the M1 type, playing an important role in wound healing and anti-inflammatory repair; however, in the late stage of chronic inflammation, the expression of the M1 type dominates, leading to neural damage. Therefore, regulating the transformation of MGl M1/M2 phenotypes is of great significance for maintaining CNS stasis (Orihuela et al., 2016; Figure 1).

## 4 Microglia polarization regulation and core mechanisms of neuroinflammation

Neurological dysfunction triggered by MG-mediated neuroinflammation may play an important role in the pathogenesis of AD. Modulating the targets of MG-mediated neuroinflammation may be an effective strategy for treating neuroinflammation in AD.

It is thus known that the occurrence and development of neuroinflammation are usually closely related to the activation of MG. The following sections will elaborate on several key targets involved in the process of MG-mediated neuroinflammation, including classical signaling pathways and emerging regulatory nodes.

### 4.1 Classical Signaling pathway

#### 4.1.1 NLRP3

**Figure 2:**
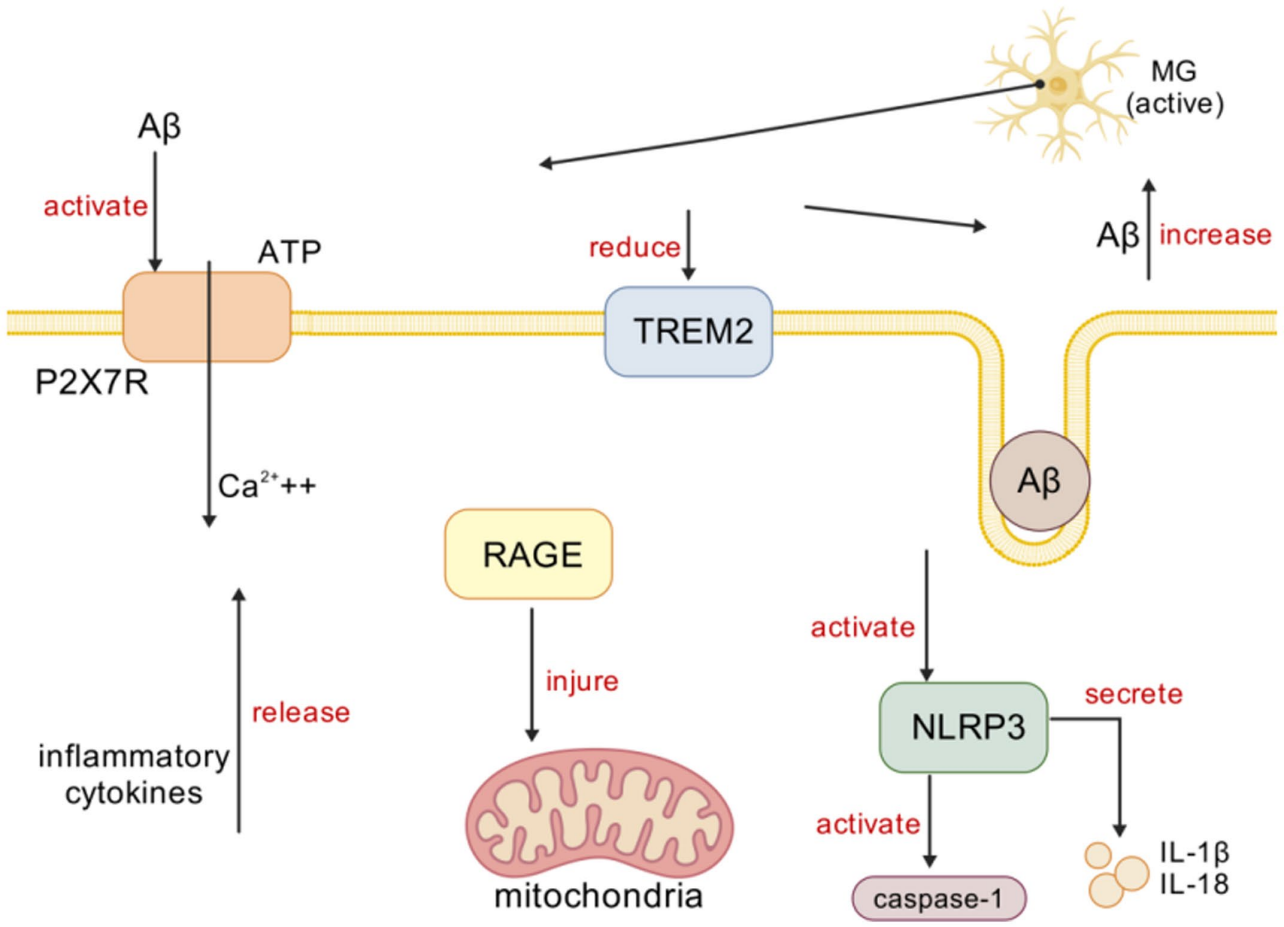
Microglia activation in AD. [Created with BioGDP.com (Jiang et al., 2025)] MG primarily interacts with targets, such as P2X7R, TREM2, NLRP3, and RAGE, in mediating inflammation. In AD, Aβ induces immune cells to release ATP, which activates P2X7R and prompts MG to release inflammatory mediators, triggering neuroinflammation. Aβ activates TREM2 signaling, promoting Aβ phagocytosis. In AD, Aβ activates NLRP3 inflammasomes, subsequently activating caspase-1 and releasing IL-1β and IL-18. Upregulation of RAGE expression in AD leads to mitochondrial damage.

In AD, Aβ, as a stimulant, activates the NOD-like receptor protein 3 (NLRP3) inflammasome. The NLRP3 inflammasome is composed of multiple cytoplasmic protein complexes (He et al., 2016), and upon activation, it activates caspase-1 and releases pro-inflammatory cytokines IL-1β and interleukin-18 (IL-18) (Kelley et al., 2019). The activation of the NLRP3 inflammasome is related to microtubule-associated protein tau (tau) pathology. For example, studies have found that loss-of-function mutations in the NLRP3 inflammasome reduce tau pathology and prevent cognitive decline in heterozygous THY-Tau22 transgenic mice (Kelley et al., 2019). The serotonin receptor antagonist and reuptake inhibitor trazodone reduces neuroinflammation mediated by MG and NLRP3 inflammasome activation, thereby alleviating memory and sleep disturbances (Ising et al., 2019). In the streptozotocin-induced Alzheimer’s disease mouse model, the NLRP3 inflammasome inhibitor MCC950 protects against pathological reactive MG by inhibiting the activation of the NLRP3 inflammasome (De Oliveira et al., 2022). Currently, modulating neuroinflammatory responses mediated by NLRP3 is being evaluated as a therapeutic target for AD (Feng et al., 2020). IL-1β, part of the innate immune response, is released through NLRP3 inflammasome activation, and the increase of IL-1β is related to Aβ plaques, tau hyperphosphorylation, and neurofibrillary tangles (He et al., 2020). Hippocampal LPS injection induces the production of IL-1β, leading to memory impairment by inducing MGl activation. IL-1β antibodies alleviate memory and cognitive impairments in 3xTg-AD mice by inhibiting nuclear factor κB (NF-κB) activation and reducing the activation of tau-related kinases, including the cyclin-dependent kinase 5/p25 (cdk5/p25), glycogen synthase kinase-3β (GSK-3β), and P38 mitogen-activated protein kinase (p38-MAPK) (Feng et al., 2020). Therefore, IL-1β antibodies are also suggested as potential therapeutic agents targeting neuroinflammation in the later stages of AD (Sheng et al., 2001; Figure 2).

#### 4.1.2 Rage

The receptor for advanced glycation end products (RAGE) is related to Aβ pathology, especially in the process of Aβ entering the brain from the periphery. In AD, the upregulation of RAGE on endothelial cells leads to an increase in brain Aβ levels (Liu et al., 2009; Abdallah et al., 2022). RAGE plays a key role in the transport of peripheral Aβ across the blood–brain barrier (BBB) (Cai et al., 2016), and studies injecting RAGE-specific IgG into transgenic (Tg)2,576 mice to block RAGE-mediated Aβ transport across the BBB have confirmed this (Deane et al., 2003). RAGE-mediated Aβ transport is associated with an increase in inflammatory cytokines (such as IL-6 and TNF-*α*). RAGE is also expressed in MG, and studies have found that its upregulation leads to mitochondrial damage, oxidative stress, and mitochondrial autophagy (Zhang et al., 2020). In addition to Aβ, RAGE interacts with multiple ligands, including damage-associated molecular patterns (DAMPs) such as high mobility group box 1 (HMGB1) (Baiguera et al., 2009). The interaction of HMGB1 with RAGE regulates immune processes by upregulating pro-inflammatory cytokines and chemokines (Harris et al., 2012). RAGE and HMGB1 are upregulated in AD, and their interaction leads to the activation of inflammatory signaling pathways, including NF-κB, and cell death (Paudel et al., 2020; Figure 2).

### 4.2 Emerging regulatory node

#### 4.2.1 P2×7R

The purinergic 2×7 receptor (P2X7R) is an ion channel present on the surface of MG and is activated by the binding of adenosine triphosphate (ATP). In AD, the P2X7R plays an important role in the activation of MG (Francistiová et al., 2020; Oliveira-Giacomelli et al., 2021; Chen et al., 2021). Studies have shown that the P2X7R is overexpressed in AD, and Aβ triggers the release of ATP from immune cells, activating the P2X7R, which, in turn, activates MG to release inflammatory cytokines, causing neuroinflammation (Martin et al., 2019; Monif et al., 2010). In hAPP-J20 mice, EGFP under the P2X7R promoter shows P2X7R up-regulation in microglia encircling Aβ plaques. This up-regulation draws MG to the plaques yet paradoxically blunts their phagocytosis. The use of the selective P2X7R antagonist glycogen synthase kinase 1482160A (GSK1482160A) can increase the migration and phagocytic ability of MG, reducing neuroinflammation (Martínez-Frailes et al., 2019). Therefore, P2X7R inhibition is considered a treatment method for reducing neuroinflammation in AD (Illes et al., 2019; Figure 2).

#### 4.2.2 TREM2

Triggering receptor expressed on myeloid cells 2 (TREM2) is a transmembrane protein receptor of the innate immune system specifically expressed in brain MG (Ellwanger et al., 2021; Hu et al., 2014) and plays an important role in activating immune responses and neuroinflammation (Haure-Mirande et al., 2022). Aβ activates TREM2 signaling, thereby promoting Aβ phagocytosis. TREM2p. R62C and p. R62H loss-of-function mutations increase Aβ plaque formation and MGl aggregation, leading to neuroinflammation. TREM2 plays different roles in the progression of AD (Yang et al., 2020). Studies in 35 different transgenic mouse models have found that, in the early stage, the knockout of TREM2 reduces the secretion of inflammatory factors, while in the late stage, the knockout of TREM2 increases the secretion of inflammatory factors (Parhizkar et al., 2019). Studies using positron emission tomography (PET) and cerebrospinal fluid examination of TREM2 have shown that increased levels of TREM2 in humans are associated with reduced levels of Aβ and tau (Karanfilian et al., 2020). In addition, studies have shown that TREM2 regulates calcium signaling in induced pluripotent stem cell-derived MG (iPSC-derived MG), affecting the directed chemotaxis of MG toward Aβ plaques (Ewers et al., 2020). Downregulation of TREM2 leads to cognitive impairment, Aβ accumulation, and neuroinflammation, increasing the release of inflammatory factors through the Toll-like receptor 4 (TLR4)-mediated MAPK signaling pathway, thereby increasing inflammation (Jairaman et al., 2022). Overexpression of TREM2 in BV-2 cells increases the Aβ clearance rate and reduces neuroinflammation (Ruganzu et al., 2022; Figure 2).

## 5 Characteristics and advantages of Chinese medicine regulation

### 5.1 Multitarget synergies

Traditional Chinese medicine classifies AD as “dementia” and “forgetfulness,” believing that the disease is located in the brain and closely related to the kidneys, heart, liver, spleen, and other organs. The basic pathogenesis of AD is considered to be a deficiency in the root and an excess in the branches, with “deficiency” mainly manifested as marrow sea deficiency and qi-blood insufficiency and “excess” mostly related to phlegm-turbidity and blood stasis. The traditional Chinese medicines currently used in clinical treatment and experimental research are mostly supplements, blood-activating agents, and phlegm-removing drugs. Therefore, studying the therapeutic and ameliorative effects of TCM on AD may offer new therapeutic hope for patients.

#### 5.1.1 Active ingredients in Chinese medicines

##### 5.1.1.1 Alkaloid

Matrine, a quinolizidine alkaloid and one of the main active components of the traditional Chinese medicine *Sophora flavescens*, was found by Li et al. to significantly reduce the activation of MG in AD mice following intraperitoneal injection of different concentrations of matrine solution. The levels of inflammatory factors, such as reactive oxygen species (ROS), TNF-*α*, IL-1β, and IL-6, in the hippocampal tissue were decreased, and the expression of two types of NADPH oxidase (NOX) was downregulated (Li et al., 2020).

##### 5.1.1.2 Saponins

Ginsenoside Rg1 can significantly reduce the activity of hippocampal neurons and the expression of pro-inflammatory factors in AD mouse models. Ginsenoside Rg1 can play a role in neuroprotection and the improvement of cognition by regulating the cytokines mediated by MG activation (Shi et al., 2019). Shi Ying investigated the use of ginsenoside Rg1 combined with piracetam for treating vascular dementia over 3 months. The treatment group supplemented with ginsenoside Rg1 capsules achieved an overall response rate of 85% (compared to 60% in the control group receiving piracetam alone, *p* < 0.05), with no adverse reactions reported. Both vascular dementia and AD are associated with excessive microglial activation. Rg1 inhibits the inflammatory phenotype of MG, reducing Aβ-induced release of TNF-*α* and IL-1β; meanwhile, piracetam blocks the damage caused by inflammatory cytokines to neural synapses. These two agents synergistically regulate microglial homeostasis, offering a reference for combined therapeutic approaches in AD (Shi et al., 2011).

Astragaloside, the main active component of the traditional Chinese medicine *Astragalus membranaceus*, can significantly improve central nervous system inflammation caused by brain injury in rats, increase the expression of peroxisome proliferator-activated receptor *γ* (PPARγ), and promote the release of neuroprotective factors (Li et al., 2021). Astragaloside has an inhibitory effect on the activity of MG in AD rat models through the mitogen-activated protein kinase 5/extracellular-signal regulated protein kinase 5 (MEK5/ERK5) signaling pathway by reducing the levels of interleukin-1 (IL-1) and TNF-α, thereby inhibiting inflammatory responses; by reducing the expression levels of glucose-regulated protein 78 (GRP78) and the C/EBP homology protein (CHOP), thereby protecting brain function; and by reducing the expression of MEK5 and ERK5 proteins, thereby playing a role in nuclear transcription regulation. It can reduce the apoptosis of nerve cells in AD rats (Fang et al., 2021; Li et al., 2018).

##### 5.1.1.3 Flavonoids

The effective component of *Epimedium*, icariin, has been proven to be an effective anti-inflammatory agent. Exploring the mechanism through which icariin regulates the polarization of MG, using inflammatory cytokines (IL-1β, IL-6, and TNF-*α*) to mark M1-type cells and anti-inflammatory factors (IL-4, IL-10, and transforming growth factor-1 (TGF-1)) to mark M2-type cells, it was found that icariin can significantly improve the swimming performance of the amyloid precursor protein/ presenilin 1 (APP/PS1) double transgenic mice in the water maze and inhibit the expression of M1-type markers and its mechanism may be through the activation of the PPARγ pathway to induce the polarization of M1 to M2. In addition, the transcription regulatory factor, nuclear factor κB (NF-κB), is also an important signal molecule for the regulation of MG activation, phenotype transformation, and neuroinflammation by traditional Chinese medicine (Wang et al., 2019).

Isoflavone components in soybeans can exert neuroprotective effects by inhibiting the activation of MG and the production and release of soluble pro-inflammatory factors after activation (Chinta et al., 2013).

Baicalin treatment can weaken the activation of MG and AST, reduce the expression of TNF-α and IL-6, and significantly improve learning and memory impairments (Chen et al., 2015).

##### 5.1.1.4 Phenolics

Curcumin can effectively inhibit the release of inflammatory factors in BV-2 cells induced by LPS, reduce the proportion of M1-type cells, and enhance cellular phagocytosis. It is speculated that curcumin may inhibit MG activation and play a phagocytic and anti-inflammatory role by targeting the TREM2-TLR4-mediated NF-κB signaling pathway (Zhang et al., 2019; Gao et al., 2019).

Resveratrol, a non-flavonoid polyphenolic compound widely present in many plants, has become a hot topic in AD research in recent years due to its significant neuroprotective and immune-modulating effects (Li, 2016). Resveratrol can inhibit the NF-κB signaling pathway in MG, effectively reduce the expression of NF-κB/IL-1β/inflammasome NLRP3, and alleviate inflammatory responses (Qi et al., 2019; Zhao et al., 2018).

##### 5.1.1.5 Terpenes

The component catalpol in *Rehmannia glutinosa* can inhibit the protein expression of TLR4 and myeloid differentiation primary response protein 88 (MyD88) induced by LPS, reduce the combination of TLR4 and LPS on the cell surface, and thus inhibit the activation of the NF-κB signaling pathway to exert anti-inflammatory and neuroprotective effects (Choi, 2019). The administration of Liuwei Dihuang pills (with prepared *Rehmannia* root as the primary ingredient) combined with donepezil for 12 weeks for treating AD of kidney yin deficiency type yielded Chinese and Western medicine efficacy rates of 73.3 and 76.7%, respectively, in the trial group, outperforming donepezil monotherapy (60 and 56.7%). Significant improvements were observed in the mini–mental state examination, Activities of Daily Living Scale scores, and TCM syndrome scores (*p* < 0.05), with no adverse reactions reported. This compound has been demonstrated to reduce neuroinflammation. Further studies should validate its potential to inhibit excessive MGI activation and reduce inflammatory cytokine release, thereby synergistically enhancing cognitive and daily living abilities with donepezil at the pathological level (Rao, 2024).

Tanshinone I can significantly reduce the production of NO, TNF-*α*, and IL-6, inhibit the NF-κB signaling pathway, achieve the activation of the M2 type, exert a neuroprotective effect, and show a dose-dependent effect (Wang et al., 2015).

The component geniposide in *Gardenia jasminoides* can inhibit the activation of MG by inhibiting the RAGE-mediated signaling pathway RAGE-ERK1/2-NF-κB, block the release of pro-inflammatory factors such as IL-1*β* and TNF-α from MG, reduce Aβ deposition, and exert neuroprotective effects (Lv et al., 2015).

##### 5.1.1.6 Phenylpropanoid

β-Asarone in *Acorus tatarinowii* can exert anti-inflammatory effects by inhibiting the production of pro-inflammatory mediators and the activation of MG through the NF-κB signaling pathway and c-Jun N-terminal kinase (JNK) signaling pathway (Lim et al., 2014).

Carotenoids:

The main component of *Crocus sativus*, saffronin, can significantly inhibit the production of inflammatory factors and reactive oxygen species induced by LPS in BV-2 cells, and its mechanism may involve inhibition of the NLRP3 and NF-κB signaling pathways, promoting the polarization of MG toward the M2 type to exert anti-inflammatory effects (Zhang et al., 2018).

The therapeutic mechanisms of active components of TCM for Alzheimer’s disease, via the modulation of microglia, are summarized in Table 1.

**Table 1:** Therapeutic mechanisms of active components of TCM for AD via the modulation of MG.

Pathway	Active ingredient	Mechanism	Literature
Alkaloid	Matrine	ROS, TNF-α, IL-1β, and IL-6↓, NOX↓	Li et al. (2020)
Saponins	Ginsenoside Rg1	TNF-α and IL-6↓	Shi et al. (2019)
	Astragaloside	PPARγ↑IL-1, TNF-α↓GRP78, CHOP↓MEK5, and ERK5↓	Fang et al. (2021), Li et al. (2021), and Li et al. (2018)
Flavonoids	Icariin	PPARγ↑	Wang et al. (2019)
	Isoflavones	NO, IL-6, mRNA, ROS↓p38 MAPK, and NF-κB↓	Chinta et al. (2013)
	Baicalin	TNF-α and IL-6↓	Chen et al. (2015)
Phenolics	Curcumin	NF-κB↓	Zhang et al. (2019) and Gao et al. (2019)
	Resveratrol	NF-κB/IL-1β/NLRP3↓	Li (2016), Qi et al. (2019), and Zhao et al. (2018)
Terpenes	catalpol	TLR4 and MyD88↓	Choi (2019)
	Tanshinone I	NO, TNF-α, IL-6↓, and NF-κB↓	Wang et al. (2015)
	Geniposide	ERK1/2, IKB, NF-KB↓IL-1β, and TNF-α↓	Lv et al. (2015)
Phenylpropanoid	β-Asarone	NF-κB↓	Lim et al. (2014)
Carotenoids	Saffronin	NLRP3 and NF-κB↓	Zhang et al. (2018)

#### 5.1.2 Chinese herbal formula

TCM formulas are characterized by multi-component, multi-target, multi-pathway, multi-system, and multi-link mechanisms in treatment.

Hei Xiao Yao San: It can regulate the inflammatory response in the hippocampus of AD rats by controlling the wingless-integrated (Wnt)/β-catenin signaling pathway and improve cognitive impairment in AD rat models (Lou et al., 2019). Lou Q used modified Hei Xiao Yao San to treat AD, with the control group receiving oral Baifukang (piracetam tablets). The treatment group demonstrated significantly superior efficacy compared to the control group (*p* < 0.05), indicating the confirmed therapeutic efficacy of modified Hei Xiao Yao San. Whether its mechanism involves regulating MG warrants further investigation (Lou et al., 2019).

Nao Ling Tang: Studies have shown that Nao Ling Tang can regulate the expression of inflammatory factors in the brain, inhibit the expression of MG in the hippocampus of rat models, and has anti-inflammatory effects (Ge, 2013).

Shao Yao Gan Cao Tang: Research has found that Shao Yao Gan Cao Tang reduces the expression of inflammatory factors TNF-*α*, IL-1β, and IL-6 in the hippocampus and cerebral cortex of mice, inhibits the aggregation of Aβ in the brain and the hyperphosphorylation of the tau protein, and exerts anti-Aβ deposition and neuroprotective effects by downregulating the expression of nucleotide-binding oligomerization domain-like receptor proteins NLRP1 and NLRP3 (Chiu et al., 2021).

Huang Lian Jie Du Tang (Ren et al., 2021; Xie, 2015): Huang Lian Jie Du Tang can inhibit the production and accumulation of Aβ and the abnormal phosphorylation of the tau protein by regulating the heat shock protein 70 (HSP70)-mediated neuroprotective mechanism, thereby suppressing the activation of AST and MG, reducing brain neuroinflammation, alleviating neuronal apoptosis, maintaining hippocampal neurons and dendritic spines, and playing a neuroprotective role (Tan, 2022). Some studies have found that gut ecological disorders are highly related to neuroinflammation caused by AD and can play an anti-neuroinflammatory role by regulating the levels of interleukin-6 and interferon-*γ* in APP/PS1 mice, thereby improving cognitive dysfunction in AD (Wang, 2022). It can also improve cognitive dysfunction in AD mouse models caused by high-fat diets by improving the microenvironment of gut flora related to bile acids and arachidonic acid metabolism, as well as by improving the expression of liver X receptors and peroxisome proliferator-activated receptors, thereby reducing neuroinflammatory responses (Gu et al., 2021).

Bu Shen Yi Sui Capsule: The mechanism of the Bu Shen Yi Sui Capsule may be related to the downregulation of microRNA-124 (miR-124) expression, upregulation of microRNA-155 (miR-155) expression, and promotion of M2-type cell polarization (Zha et al., 2021).

Ba Zi Bu Shen Capsule (BZBS): It has anti-aging effects and is effective in enhancing memory. Some studies have found that it improves cognitive dysfunction by inhibiting cellular senescence and MGl activation (Ji et al., 2024).

Bu Shen Yi Zhi Anti-Aging Formula: It may improve cognitive function in APP/PS1 Tg mice by inhibiting the TLR4/Myd88/NF-κB signaling pathway, suppressing the activation of brain MG, and promoting the transformation of M1-type MG to the M2 type (Wang et al., 2022).

Xiao Chai Hu Tang: It may inhibit the polarization of MG by inhibiting the phosphorylation of the Janus kinase/signal transducer and activator of transcription (JAK2/STAT3) pathway, reducing central inflammation, and exerting an antidepressant effect (Zhang et al., 2021).

Dang Gui Shao Yao San (Li et al., 2023): It has been found to inhibit the activation of MG by regulating the TLR4/MyD88/NF-κB signaling pathway, thereby inhibiting neuroinflammatory responses and playing a neuroprotective role. The specific manifestations include a significant decrease in the mRNA expression levels of inflammatory factors IL-1*β*, IL-6, and TNF-*α*; a significant reduction in the expression levels of TLR4 and MyD88, a significant decrease in the ratio of p-NF-κB p65 to NF-κB p65, and a significant reduction in the expression of NF-κB p65 in the nucleus.

Tiao Xin Fang (Sun et al., 2003) and Kai Xin San (Guo et al., 2019a; Guo et al., 2019b; Luo et al., 2020): They can inhibit the activation of MG; significantly reduce the expression of inflammatory cytokines, such as NF-κB, IL-1β, IL-6, IL-8, and TNF-α, in the brain tissue of AD animal models; decrease the expression level of the β-amyloid precursor protein; alleviate neuronal damage; and significantly improve the learning and memory ability of AD animal models.

The therapeutic mechanisms of Chinese medicine compound prescriptions against Alzheimer’s disease via the modulation of microglia are summarized in Table 2.

**Table 2:** Therapeutic mechanisms of chinese medicine compound prescriptions against AD via the modulation of MG.

Chinese herbal formula	Mechanism	Literature
Hei Xiao Yao San	NOX2/ROS/NF-κB↓NOX2, NF-κB, IKBα↑ROS, MDA↑IL-6, IL-8, and TNF-α↑	Lou et al. (2019)
Nao Ling Tang	OX-42, IL-6↓	Ge (2013)
Shao Yao Gan Cao Tang	IL-6, TNF-α, IL-1β, and COX-2↓Aβ↓	Chiu et al. (2021)
Huang Lian Jie Du Tang	Aβ and Tau↓	Ren et al. (2021), Xie (2015), Tan (2022), Wang (2022), and Gu et al. (2021)
Bu Shen Yi Sui Capsule	miR-124↓miR-155↑	Zha et al. (2021)
Ba Zi Bu Shen Capsule (BZBS)	p16INK4a, Iba1↓PCNA, PSD95↑ Iba1, CD11b↓Arg1, CD206, Trem2, Ym1, Fizz1, and PSD95↑	Ji et al. (2024)
Bu Shen Yi Zhi Anti-Aging Formula	TLR4/Myd88/NF-κB↓	Wang et al. (2022)
Xiao Chai Hu Tang	JAK2/STAT3↓	Zhang et al. (2021)
Dang Gui Shao Yao San	IL-1β, IL-6, TNF-α mRNA↓TLR4, and MyD88↓p-NF-κB p65/NF-κB p65↓NF-κB p65↓	Li et al. (2023)
Tiao Xin Fang and Kai Xin San	NF-κB, IL-1β, IL-6, IL-8, and TNF-α↓	Sun et al. (2003), Guo et al. (2019a), Guo et al. (2019b), and Luo et al. (2020)

### 5.2 Advantages of overall regulation

TCM exhibits unique systems biology characteristics in regulating MG polarization, and its multi-component synergy, dynamic adaptability, and holistic regulation show unique advantages in the prevention and treatment of AD.

Chinese medicine can regulate MG polarization through a synergistic network of multi-component, multi-target, and multi-pathway mechanisms in the prevention and treatment of AD. The complex pharmacological mechanism of TCM formulations can create target complementarity and signaling pathway synergy to achieve MG metabolic reprogramming. For example, baicalin reduces the expression of the M1-type marker by inhibiting the TLR4/NF-κB pathway (Jin et al., 2019), whereas ginsenoside Rg1 promotes the production of the M2-type marker by regulating the GATA binding protein 4 / phosphodiesterase 4A/ phosphatidylinositol 3-kinase/ protein kinase B (GATA4/PDE4A/PI3K/AKT) axis (Fang et al., 2025), resulting in bidirectional regulation through pro-inflammatory inhibition and anti-inflammatory activation. Huanglian Xieyu Tang may improve AD by synergistically regulating brain and intestinal functions and remodeling the peripheral microenvironment (Gu et al., 2021).

TCM has dynamic regulatory properties that enable it to adapt to the stages of AD pathology, heterogeneity of the brain regions, and rhythmic synchronization. In the early stage of AD (Aβ deposition stage), blood-activating and blood-stasis-removing traditional Chinese medicines preferentially inhibit M1 polarization. In the tau pathological stage, kidney-supplementing and essence-filling traditional Chinese medicines enhance the phagocytosis and elimination function of M2, reflecting the temporal and spatial characteristics of the “staged treatment” (Dang et al., 2024).

Chinese medicine improves the microenvironment of neuroinflammation through multi-organ coordination and multi-dimensional regulation, breaks through the traditional single-target intervention mode, and establishes a regulatory system centered on “MG regulation of neuroinflammation,” which regulates AD neuroinflammation in a multi-dimensional way.

## 6 Conclusion and prospects

AD is a serious threat to human health, with a current lack of effective clinical treatments highlighting the urgent need for intensified drug development research. In AD progression, MG are activated to release cytokines (TNF-*α*, IL-1β, IL-6) and ROS, which exacerbate neuronal damage, impair synaptic function, and lead to cognitive decline. Activated MG also trigger neuronal apoptosis and death, worsening cognitive impairments in AD patients. The classical signaling pathway and emerging regulatory nodes, such as P2X7R, TREM2, NLRP3, and RAGE, are involved in MG activation, driving neuroinflammation and worsening AD. Therefore, MG-mediated neuroinflammation plays a key role in AD pathogenesis. TCM shows potential in treating AD by inhibiting MG activation, promoting the M2 phenotype, and regulating receptor expression, thereby reducing neuroinflammation, protecting neurons, and improving cognitive deficits through multiple pathways and targets. TCM’s rich clinical experience, diverse therapeutic approaches, minimal side effects, and holistic concept make it a promising option for AD treatment.

However, the complex pathogenesis of AD and the intricate mechanisms of TCM pose challenges in developing targeted drugs and establishing efficacy standards. Future research should focus on elucidating the role of MG in neuroinflammation and neuronal damage, as well as their interactions with other cell types. Developing targeted drugs to modulate key molecules and pathways involved in MGl activation could offer new therapeutic strategies for AD.

Although traditional Chinese medicine (TCM) shows tremendous potential in regulating microglia for treating Alzheimer’s disease, safety concerns in its clinical application remain an unavoidable critical issue and a barrier that must be overcome for successful translation. Currently, challenges in using TCM to modulate microglia for treating Alzheimer’s neuroinflammation include the following: unclear quantitative analysis of synergistic effects among multiple TCM components, difficulty in dynamically monitoring microglial polarization states, limited application of spatiotemporal specificity in TCM interventions for personalized treatment plans, lack of high-quality clinical trials, challenges in standardizing TCM formulas, and unresolved issues regarding the pharmacokinetics and BBB permeability of active ingredients. Moreover, the vast majority of evidence comes from preclinical models, which struggle to fully replicate the complex physiological environment of the human body and its response to multi-component TCM systems, thereby limiting safety predictions. Additionally, systematic pharmacokinetic studies, tissue distribution characteristics, and potential organ toxicity data for active TCM components remain severely lacking. Moreover, potential interactions among multiple components within TCM formulas and between TCM and conventional chemical drugs represent an underexplored risk domain.

To advance TCM in AD treatment, clinical research should be intensified to establish clear efficacy assessments and accumulate large-scale clinical data. Integrating TCM with modern medicine could create a synergistic treatment model, combining evidence-based and holistic approaches with modern diagnostics and targeted therapies, which offers more effective options for AD patients. The goal is to design Chinese medicine carriers targeting MG and optimize intelligent drug delivery systems. It is important to promote research on the role of the “ruler, minister, auxiliary, and envoy” principle in regulating the MG regulatory network. Establishing the relationship between MG biotyping and traditional Chinese medicine intervention at different stages of AD is also crucial.

## Glossary

Aβ: Amyloid Beta Peptide
NFTs: Neurofibrillary Tangles
MG: Microglia
AD: Alzheimer’s Disease
TCM: Traditional Chinese Medicine
CNS: Central Nervous System
MCI: Mild Cognitive Impairment
LPS: Lipopolysaccharide
IFN-γ: Interferon-Γ
TNF-α: Tumor Necrosis Factor-Α
IL-1β: Interleukin-1Β
IL-6: Interleukin-6
NO: Nitric Oxide
CD16/32: Cluster of Differentiation 16/32
IL-10: Interleukin-10
IL-4: Interleukin-4
BDNF: Brain-Derived Neurotrophic Factor
IGF-1: Insulin-Like Growth Factor 1
SOCS3: Suppressor of Cytokine Signaling 3
CD163: Cluster of Differentiation 163
NLRP3: NOD-Like Receptor Protein 3
IL-18: Interleukin-18
tau: Microtubule-Associated Protein Tau
cdk5/p25: Cyclin-Dependent Kinase 5/P25
GSK-3β: Glycogen Synthase Kinase-3Β
p38-MAPK: P38 Mitogen-Activated Protein Kinase
RAGE: The Receptor of Advanced Glycation Endproducts
BBB: Blood–Brain Barrier
DAMPs: Damage Associated Molecular Patterns
HMGB1: High Mobility Group Box-1 Protein
P2X7R: Purinergic 2×7 Receptor
ATP: Adenosine Triphosphate
hAPP: Human Amyloid Precursor Protein
EGFP: Enhanced Green Fluorescent Protein
GSK1482160A: Glycogen Synthase Kinase 1482160A
TREM2: Triggering Receptor Expressed On Myeloidcells 2
PET: Positron Emission Tomography
TLR4: Toll-Like Receptor 4
ROS: Reactive Oxygen Species
NOX: NADPH Oxidase
PPARγ: Peroxisome Proliferator Activated Receptor Γ
MEK5/ERK5: Mitogen-Activated Protein Kinase Kinase 5/Extracellular Regulated Protein Kinases 5
IL-1: Interleukin-1
GRP78: Glucose Regulated Protein 78
CHOP: C/EBP Homology Protein
TGF-1: Transforming Growth Factor-1
APP/PS1: Amyloid Precursor Protein/ Presenilin 1
NF-κB: Nuclear Factor Kappa-B
MyD88: Myeloid Differentiation Primary Response Protein 88
JNK: C-Jun N-Terminal Kinase
Wnt: Wingless-Integrated
NLRP1: Nucleotide- Binding Oligomerization Domain
HSP70: Heat shock protein 70
AST: Astrocyte
miR-124: Microrna-124
miR-155: Microrna-155
Tg: Transgene
JAK2/STAT3: Janus Kinase/Signal Transducer and Activator of transcription
GATA4: GATA Binding Protein 4
PDE4A: Phosphodiesterase 4A
PI3K: Phosphatidylinositol-3-Kinase
AKT: Protein Kinase B
